# Travel burden associated with rare cancers: The example of Merkel cell carcinoma

**DOI:** 10.1002/cam4.2085

**Published:** 2019-04-05

**Authors:** Rahul Jain, Joseph Menzin, Kristina Lachance, Patrick McBee, Hemant Phatak, Paul T. Nghiem

**Affiliations:** ^1^ Boston Health Economics BHE Boston Massachusetts; ^2^ Division of Dermatology, Department of Medicine University of Washington Seattle Washington; ^3^ EMD Serono, Inc. Rockland Massachusetts; ^4^ Clinical Research Division Fred Hutchinson Cancer Research Center Seattle Washington

**Keywords:** cancer management, clinical observations

## Abstract

**Background:**

There are limited data on the travel burden for cancer patients with rare tumor types, such as Merkel cell carcinoma (MCC).

**Objective:**

The objective of this study was to understand the travel burden of MCC patients.

**Methods:**

This study used data from an MCC registry at the Seattle Cancer Care Alliance (SCCA). All MCC patients enrolled at SCCA with a valid 3‐digit ZIP code were included. Patients were followed up from January 1, 2012 until their last follow‐up, death, or end of data (January 1, 2017). Travel burden was measured by one‐way travel distance to SCCA from each patient's 3‐digit ZIP code. Patient demographics, tumor characteristics, and follow‐up visit were evaluated and stratified by one‐way driving distance of ≤300 and >300 miles.

**Results:**

A total of 391 MCC patients were included (68% men, mean age = 67 years [±SD = ±11 years], 67% residing in the West, and 70% white). At diagnosis, 53% of the patients had Stage III or IV MCC. Mean one‐way distance traveled by patients was 1,137 (median: 813) miles, and 57% of patients traveled >300 miles. Compared to patients who traveled ≤300 miles, those who traveled >300 miles were more likely to be <70 years old (46% vs 65%; *P* < 0.001), were diagnosed with advanced stage (III or IV) MCC (46% vs 59%; *P* = 0.01), had shorter follow‐up in the cancer registry (mean: 509 vs 212 days; *P* < 0.001), and had fewer visits during follow‐up (mean: 5.2 vs 2.5; *P* < 0.001).

**Conclusions:**

In this single cancer center study, the majority of MCC patients trav‐eled long distances to receive expert care. Longer travel distances appeared to be associated with younger age, a more advanced stage of cancer at study entry and fewer in‐clinic visits, suggesting that travel burden may impact timely and adequate patient care for this rare cancer.

## 1 INTRODUCTION

In the United States (US), diseases with prevalence of less than 750 cases per million people are classified as rare.^1^ There are over 6,000 diseases that are designated as rare.^2^ The definition of rare cancers, however, is based on incidence rates rather than prevalence rates since prevalence can be a misleading indicator of rarity for disorders that occur infrequently.^3^ In the US, rare cancers are defined as those with an incidence rate of between 60 and 150 cases per million individuals per year.^4^, ^5^ Currently, more than 500 cancer types are designated as rare,^6^ constituting approximately 20% of cancer diagnoses in the US.^4^, ^5^

Rare cancers pose a significant burden on the patient population. Outcomes in patients with rare cancers are worse than those with more common tumor types.^7^ A variety of issues, including difficulty or delay in diagnosis, misdiagnosis, limited access to centers with clinical expertise, less effective standard treatments, and inadequate funding for pre‐clinical and clinical research programs, contribute toward poor health outcomes in this population.^3^, ^5^

Moreover, patients with rare cancers frequently face a travel burden, including additional travel time and costs, likely influencing access to both adequate and timely diagnosis and treatment services.^8^ Previous literature has demonstrated that high travel burden is associated with a delay in diagnosis, leading to more advanced disease at diagnosis, inappropriate treatment, worse prognosis, and lower quality of life.^8^ In the case of rare cancers, diagnosis is particularly challenging, resulting in numerous visits to centers with clinical expertise. Since the number of such specialized centers for rare cancers is limited, patients may travel more often and farther relative to patients with other common cancers.^3^, ^5^

Merkel cell carcinoma (MCC) is a rare, aggressive, lethal form of skin cancer, often appearing as a red, purple, or skin colored tumor nodule which is often misdiagnosed as a cyst.^9^ In the US, approximately 80% of MCC cases are causally linked to the Merkel cell polyomavirus (MCPyV), while the remaining 20% of cases are caused by extensive UV mutations.^10^, ^11^ The incidence rate of MCC is 7.9 per one million patients per year.^12^ In treating MCC, there are only a few specialty centers across the US; 55 specialists work out of 41 centers in 22 states.^13^ Patients are often referred to these centers for diagnosis, treatment, and regular check‐ups to ensure optimal care and management of MCC. However, the scarcity of specialty centers may pose a significant burden on both the patients and their families through increased travel burden to such centers, decreased access, and/or increased cost of treatment. The issue of travel burden has taken center‐stage lately, with emphasis on patient‐centric approaches to care and increasing dialogue about financial toxicity.^14^

The aim of this study was to evaluate the travel burden associated with MCC using data from a Seattle‐based cancer registry.

## 2 MATERIALS AND METHODS

### 2.1 Study site and data source

To understand the travel burden faced by MCC patients, a retrospective, single cohort study was conducted using a large MCC registry maintained by a Seattle‐based team from the University of Washington and the Seattle Cancer Care Alliance (SCCA). This team operates in one of the few large volume MCC specialty centers and typically sees approximately 170 unique MCC patients annually. Patients from all 50 states have been seen at this center. Since there are few prospective clinical studies of MCC, and accruing patient cases is challenging, as a first step, the SCCA researchers created a repository of MCC patients to effectively understand and address these patients’ needs. This repository collected clinical data, blood samples, and biopsy and archival tissues. Specifically, this repository collected data on age, gender, race, ethnicity, 3‐digit ZIP code of the place of residence, tumor characteristics, immune status, previous and subsequent therapy exposure, lesions targeted, treatment dates, scan/physician reports, previous response to treatment, acute and late toxicity, imaging data, and follow‐up visits to SCCA and mortality.^15^

### 2.2 Patient selection and follow‐up

To be included in the analysis, patients were required to have a valid 3‐digit ZIP code of residence, SCCA as the primary enrollment site, and an enrollment date any time after January 1, 2012. Patients meeting the criteria above were followed up from the enrollment date until the database cut‐off date (January 1, 2017), death, or loss of follow‐up, whichever occurred first.

### 2.3 Study measures and stratification

Patient characteristics, such as age, gender, race, and cancer stage at diagnosis, were evaluated at baseline. Region was based on the 3‐digit ZIP code of residence and categorized based on the Census Regions and Divisions of the US. Additionally, the number of follow‐up visits and average length of follow‐up were summarized for each patient. The main outcome of interest was travel burden, which was measured as a travel segment (ie, one‐way trip) with distance imputed based on patients’ 3‐digit ZIP codes and the location of the SCCA. First, all 5‐digit ZIP codes within the 3‐digit ZIP code were identified, and the travel distance between the center of each 5‐digit ZIP code and the SCCA was calculated. For each 3‐digit ZIP code, the weighted average of travel distance was calculated; the weights were the proportion of the 3‐digit ZIP code's population residing within each 5‐digit ZIP code.

After determining the travel distance for each patient based on their 3‐digit ZIP code, the cohort was stratified into two groups: those traveling more than 300 miles (one way), and those traveling less than or equal to 300 miles (one way). The cut‐off of 300 miles was arbitrarily chosen and is approximately equivalent to 5‐6 hours of driving time.

Patients residing within 300 miles from the SCCA were assumed to drive to the center, and their travel time was determined using Google Maps. For patients traveling more than 300 miles, minimum flight time was obtained by calculating the time from the center of each 5‐digit ZIP code within the 3‐digit ZIP code, and then for each 3‐digit ZIP code, weighted average of travel time was calculated where, as before, the weights were the proportion of the 3‐digit ZIP code's population residing within each 5‐digit ZIP code.

The travel costs for each subgroup were calculated using one of two rates, accounting only for the direct cost of travel. For patients assumed to be driving (≤300 miles), the rate was equal to $0.54 per mile, which is the standard mileage rate for 2016 according to the Internal Revenue Service.^16^ For patients flying (>300 miles), the cost per mile was equal to the system passenger yield per equivalent seat mile, $0.15, obtained from the Massachusetts Institute of Technology website.^17^ Using the appropriate rate and travel distance, we calculated costs for each round‐trip observed in the database for each patient.

### 2.4 Statistical analysis

All demographic and baseline clinical characteristics and study measures of interest were described with univariate statistics. Mean, standard deviation (SD), median, and relative frequency and percentage were calculated and presented. Patients were classified according to whether they traveled ≤300 miles or >300 miles. Two‐sided Student's *t* tests were used to test continuous variables, and Chi‐Square tests were used to test categorical variables. All statistical analyses were performed using SAS version 9.4 (SAS Institute, Cary, NC).

## 3 RESULTS

### 3.1 Baseline characteristics and follow‐up

There were 1268 patients enrolled in the registry at the end of the follow‐up period. Out of these patients, 644 patients were registered at the SCCA and 551 patients had valid 3‐digit ZIP code information. Of these 551 patients, 391 patients were enrolled after January 1, 2012 and thus, formed the study population. These patients were predominantly men (68%), older (mean age = 67 years [±SD = ±11 years]), white (70%), and residing in the West (67%) (Table 1). Based on cancer stage at diagnosis, approximately 36% of the patients had Stage I MCC, 11% had Stage II MCC, 45% had Stage III MCC, and 8% had progressed to Stage IV MCC. The most common primary tumor site was head and neck (36%) and the least common was buttocks (5%). Other tumor sites included trunk (7%), upper limb (21%) and lower limb (15%) (Table 2). The average length of follow‐up for all patients was 342 days, and patients on average visited the SCCA approximately 4 times during the entire follow‐up period (Table 3).

**Table 1:** Baseline demographic characteristics

n	All patients	≤300 Miles	>300 Miles
	391	167	224
Sex, n (%)			
Female	126 (32.2)	62 (37.1)	64 (28.6)
Male	265 (67.8)	105 (62.9)	160 (71.4)
Age			
Mean [±SD]	67.2 [±10.8]	70.1 [±10.9]	65 [±10.2]
Age group, n (%)			
<40	4 (1.0)	1 (0.6)	3 (1.3)
40‐49	11 (2.8)	1 (0.6)	10 (4.5)
50‐59	74 (18.9)	26 (15.6)	48 (21.4)
60‐69	133 (34.0)	49 (29.3)	84 (37.5)
70‐79	124 (31.7)	57 (34.1)	67 (29.9)
80‐89	45 (11.5)	33 (19.8)	12 (5.4)
Region, n (%)			
Midwest	21 (5.4)	0 (0.0)	21 (9.4)
Northeast	15 (3.8)	0 (0.0)	15 (6.7)
South	94 (24.0)	0 (0.0)	94 (42.0)
West	261 (66.8)	167 (100)	94 (42.0)
Race, n (%)			
Asian	3 (0.8)	2 (1.2)	1 (0.4)
Black	1 (0.3)	1 (0.6)	0 (0.0)
White	273 (69.8)	124 (74.3)	149 (66.5)
Other	8 (2.0)	4 (2.4)	4 (1.8)
Unknown	106 (27.1)	36 (21.6)	70 (31.3)

**Table 2:** Baseline clinical characteristics

	All patients	≤300 Miles	>300 Miles
		
Primary tumor site, n (%)			
n	391	167	224
Head and neck	139 (35.6)	66 (40.0)	73 (32.6)
Trunk	26 (6.7)	11 (6.6)	15 (6.7)
Buttocks	19 (4.9)	7 (4.2)	12 (5.4)
Upper limb	83 (21.3)	47 (28.3)	36 (16.1)
Lower limb	58 (14.9)	17 (10.2)	41 (18.3)
Unknown^a^	65 (16.7)	18 (10.8)	47 (21.0)
Stage at diagnosis, n (%)			
n	369	160	209
Stage I	133 (36.0)	65 (40.6)	68 (32.5)
Stage II	39 (10.6)	21 (13.1)	18 (8.6)
Stage III	167 (45.3)	69 (43.1)	98 (46.9)
Stage IV	30 (8.1)	5 (3.1)	25 (12.0)

a No primary tumor observed; first MCC discovered in the lymph nodes.

**Table 3:** Follow‐up

	All patients	≤300 Miles	>300 Miles
Number of follow‐up visits (all patients)			
n	375	165	210
Mean [±SD]	3.7 [±3.8]	5.2 [±4.5]	2.5 [±2.5]
Minimum	1	1	1
P25	1	2	1
Median	2	4	2
P75	5	7	3
Maximum	25	25	17
Length of follow‐up (all patients) (days)			
n	375	165	210
Mean [±SD]	342.3 [±486.5]	508.5 [±567.9]	211.7 [±362.7]
Minimum	0	0	0
P25	0	28	0
Median	98	252	10.0
P75	546	882	329
Maximum	2394	2394	2036

a P25, 25th percentile; P75, 75th percentile

### 3.2 Travel burden

Patients traveled a mean distance of 1137 miles per one‐way trip segment ([±SD = ±1124.3 miles]; median = 813.0 miles). Forty‐three percent of patients traveled ≤300 miles, and the rest traveled more than 300 miles (57%). For those patients who traveled ≤300 miles, the average travel distance was 74 miles ([±SD = ±72.3 miles]; median = 57.5 miles), while those who traveled >300 miles averaged 1931 miles ([±SD = ±852.9 miles]; median = 2063.7 miles) per one‐way trip segments. Furthermore, patients with travel distance >300 miles spent on average 5.8 hours longer per one‐way trip segment traveling to the SCCA than patients with travel distance ≤300 miles.

There was also a significant difference in the cost associated with the round‐trip travel for the two groups: the estimated cost for patients traveling ≤300 miles was approximately $79 [±SD = ±$78.1], while for patients traveling >300 miles, the approximate cost was $579 [±SD = ±$255.9]. Accounting for multiple round‐trips during the follow‐up period, the travel cost was $1448 for patients traveling >300 miles and $416 for those traveling ≤300 miles.

### 3.3 Stratified by travel burden

Compared to patients who traveled ≤300 miles per one‐way segment, those who traveled >300 miles were more likely to be <70 years of age (46% vs 65%; *P* < 0.001) and diagnosed at Stage III or IV (46% vs 59%; *P* = 0.01, Table 2). The length of follow‐up for patients with travel distance >300 miles was 212 days ([±SD = ±362.7 days]; median = 10.0 days), which was significantly lower (*P* < 0.001) than for patients traveling ≤300 miles, whose mean length of follow‐up was 509 days ([±SD = ±567.9]; median = 252.0 days, Table 3). Relative to patients who traveled ≤300 miles, those who traveled >300 miles had fewer number of visits to SCCA during the follow‐up period (mean: 5.2 vs 2.5; *P* < 0.001, Table 3) (Table 4).

**Table 4:** Travel burden

Characteristics	All patients	≤300 Miles	>300 Miles
		
Travel (driving) distance per one‐way travel segment (miles)			
n	391	167	224
Mean [±SD]	1137.4 [±1124.3]	73.6 [±72.3]	1930.5 [±852.9]
Minimum	8.3	8.3	327.4
P25	61.2	22.1	1184.0
Median	813.0	57.5	2063.7
P75	2176.9	83.6	2745.2
Maximum	3305.7	295.9	3305.7
Driving time per one‐way travel segment (hours)^a^			
n	N/A	167	N/A
Mean [±SD]	N/A	1.3 [±1.0]	N/A
Minimum	N/A	0.3	N/A
P25	N/A	0.6	N/A
Median	N/A	1.1	N/A
P75	N/A	1.6	N/A
Maximum	N/A	4.6	N/A
Flying time per one‐way travel segment (hours)^a^			
n	N/A	N/A	224
Mean [±SD]	N/A	N/A	7.1 [±1.8]
Minimum	N/A	N/A	3.6
P25	N/A	N/A	5.3
Median	N/A	N/A	7.3
P75	N/A	N/A	8.7
Maximum	N/A	N/A	10.1

Driving time per one‐way travel segment was calculated for one‐way travel segments ≤300 miles, flying time per one‐way travel segment was calculated for one‐way travel segments >300 miles. N/A, not applicable; P25, 25th percentile; P75, 75th percentile.

## 4 DISCUSSION

In this single center study, the burden of traveling to the cancer center for MCC patients was positively associated with more advanced cancer at study entry, a shorter follow‐up period, and fewer in‐clinic visits. Previous studies have analyzed the travel burden faced by patients with more common forms of cancer. One such study, evaluating the travel burden of colon cancer patients using the National Cancer Database from 2003 through 2010, found that only 3.9% of patients traveled more than 50 miles for diagnosis or treatment.^18^ Similarly, another study, examining the relationship between travel burden, timely diagnosis, and treatment among breast, colorectal, and lung cancer patients, found that only 18.9%‐22.2% of patients traveled more than 25 miles for diagnosis or treatment.^19^ In contrast, for a rare cancer such as MCC, we found that patients traveled an average of 1137 miles per one‐way trip segment, with nearly 60% of the patients traveling over 300 miles one‐way. These results are important and reflect a potential problem of limited access to centers with clinical expertise.

Furthermore, in line with previous research, our study found that increased travel burden was associated with diagnosis at a later stage of cancer.^8^ In one study, the diagnosis of cancer at an earlier stage allowed for less invasive treatments, as well as less treatment altogether, and therefore, lower incurred healthcare costs.^20^ Non‐medical financial costs and high travel burden are acknowledged by the President's Cancer Panel as key barriers to health care access,^21^ and are likely to be more pronounced in more underserved populations, acting as a barrier to access to care.^22^

It may be possible to reduce the travel burden associated with long travel distances to specialized centers for patients with MCC. For example, telemedicine may be used more commonly to bridge travel distances, allowing patients with rare forms of cancer to be treated near their place of residence.^23^ A major barrier to better access to expert centers through telemedicine is the lack of mechanisms for reimbursement in the current health care system. Moreover, the availability of new immuno‐oncology treatments, such as avelumab (anti PD‐L1) which was approved for use by the US Food and Drug Administration (FDA) in March 2017,^24^ pembrolizumab which was approved by the FDA in December 2018,^25^ or other immunotherapies offered through clinical trials, could allow for some visits to take place at community oncology practices, as opposed to only at specialized centers, and may help reduce the high travel burden experienced by these patients, although this remains to be seen.

### 4.1 Limitations

Although the results support that patients with rare cancers, such as MCC, face a significant travel burden, which in turn appears to be associated with later stage at diagnosis and shorter follow‐up, certain limitations must be considered. First, the present study was conducted at a single center, decreasing the generalizability of the results to all MCC patients. Second, details concerning the mode of travel were not being collected in the registry. The travel distance, time, and cost were all inferred from the 3‐digit ZIP code, leading to increased uncertainty in the estimate. The database lacked information regarding whether the patient traveled for each encounter or stayed near SCCA for evaluation or treatment. Additionally, the analysis did not evaluate indirect costs faced by the patients and their families, as the registry did not collect information on whether the patients traveled with caregivers. Therefore, future research that incorporates more detailed information, such as caregiver costs and more accurate residence information, about patients’ trips to specialized centers like SCCA is likely to provide a better understanding of the real travel burden in rare cancers.

## 5 CONCLUSION

In this single cancer center study, the majority of patients with a rare cancer, MCC, traveled long distances to receive expert care. Longer travel distances appeared to be associated with younger age and a more advanced stage of cancer at study entry. These patients also tended to have fewer in‐clinic visits, suggesting that travel burden may impact timely and adequate patient care for this rare disease.

## CONFLICT OF INTEREST

Dr Jain is employed by BHE, a research consultancy which was funded by EMD Serono, Inc. for work on this study. Dr Menzin is employed by BHE, a research consultancy which was funded by EMD Serono, Inc. for work on this study. Ms Lachance reports no conflicts of interest to disclose. Mr McBee is employed by BHE, a research consultancy which was funded by EMD Serono, Inc. for work on this study. Dr Phatak is employed by EMD Serono, Inc. Dr. Nghiem reports grants and personal fees from EMD Serono, Inc., grants and personal fees from Merck, Sharp and Dohme, personal fees from Pfizer, Inc, personal fees from Sanofi, personal fees from Genzyme, personal fees from Regeneron, during the conduct of the study; in addition, Dr. Nghiem has Ownership Interest (intellectual property) pending at the University of Washington & Fred Hutch Cancer Research Center.
